# Combination of IL-34 and AFP improves the diagnostic value during the development of HBV related hepatocellular carcinoma

**DOI:** 10.1007/s10238-022-00810-7

**Published:** 2022-03-28

**Authors:** Kehui Liu, Yezhou Ding, Yun Wang, Qingqing Zhao, Lei Yan, Jingdong Xie, Yunye Liu, Qing Xie, Wei Cai, Shisan Bao, Hui Wang

**Affiliations:** 1 Department of Infectious Diseases, Ruijin Hospital, Shanghai Jiao Tong University School of Medicine, Shanghai, 200025 China; 2 Department of Infectious Diseases, Ruijin Hospital North, Shanghai Jiao Tong University School of Medicine, Shanghai, 201801 China

**Keywords:** HBV related HCC, IL-34, Alpha-fetoprotein, Diagnostic biomarker

## Abstract

IL-34 involves in host immunity regulated carcinogenesis. Alpha-fetoprotein (AFP) is related to the development of HCC. We explored if combination of IL-34 and APF could improve the diagnostic value in HBV related hepatocellular carcinoma (HBV-HCC). Serum was obtained from HBV patients or healthy control. Liver tissue was obtained from liver biopsy in CHB, HBV related cirrhosis patients or curative resection in HBV-HCC patients. Serum IL-34 and MCSF, or intrahepatic IL-34, MCSF and CD68^+^ tumor associate macrophages (TAMs) were determined using ELISA or immunohistochemistry. Serum IL-34 was 1.7, 1.3 or 2.3-fold higher in HBV-HCC than that of CHB, HBV related cirrhosis or healthy control, which was inhibited following trans-hepatic arterial chemoembolization (TACE) in HBV-HCC patients. Intra-hepatic IL-34 was higher in HBV-HCC than that of the other three groups. Intra-hepatic IL-34 was associated with high HBV-DNA, HBeAg^−^, poor differentiation and small tumor size of HBV-HCC patients. Intra-hepatic TAMs in HBV-HCC were increased 1.7 or 1.3-fold, compared to that from CHB or HBV-cirrhosis patients. Intra-hepatic TAMs were associated with high HBV-DNA, high tumor differentiation, small tumor size, abnormal AFP and more tumor number. AFP plus serum IL-34, showed the highest AUC (0.837) with sensitivity (0.632) and highest specificity (0.931), suggesting that AFP plus IL-34 enhances the reliability for prediction of the development of HBV-HCC among CHB patients. Circulating and intra-hepatic IL-34 was upregulated gradually in HBV disease progression from CHB, cirrhosis and HCC. IL-34 may be used as a diagnostic biomarker and potential therapeutic target for the management of HBV-HCC.

**Supplementary Information:**

The online version contains supplementary material available at 10.1007/s10238-022-00810-7.

## Introduction

Hepatitis B virus (HBV) infection, a major health problem worldwide [1], is one of the major causes of hepatocellular carcinoma (HCC) [2]. HBV related HCC (HBV-HCC) is a common primary liver cancer with high mortality, high recurrence, but low post-operative survival rate, predominantly due to later diagnosis. Therefore, it is critically important to improve the detection of HCC with reliable sensitivity and specificity, which could have a significant impact on the management of HBV-HCC.

It is well known that host immunity plays a critical role in carcinogenesis [3]. Tumor microenvironment, including tumor cells, macrophages, cytokines and activated endothelial cells, plays an important role in tumor development [4]. Tumor associated macrophages (TAMs) regulate the microenvironment [5], but the role of TAMs is controversial. TAMs promotes tumor invasion, formation of blood vessels and lymphatic vessels and migration of tumor cells [6, 7], via enhancing immuno-suppression environment [8]. Concurrently, TAMs have also been linked to inhibiting cancer growth and metastasis [9]. Such discrepancy may be related to the differential polarization of macrophages during their maturation, i.e., namely classical activated M1 macrophages and alternatively activated M2 macrophages based on the surface biomarkers and the functionalities [5]. The differential polarization of macrophages is perhaps due to different microenvironments in different regions and/or in different individuals, mediated by different regulators [10].

IL-34, a member of interleukin 1 family, is produced by a wide range of cells, including macrophages, fibroblasts and hepatocytes [11, 12]. IL-34 promotes differentiation, proliferation and survival of mononuclear cells via binding to CSF-1R [13]. Dysregulation of IL-34 is involved in many diseases [14], including inflammatory bowel disease [15], rheumatoid arthritis [16], chronic heart failure [17] and ischemia/reperfusion injury-incited acute kidney injury [18]. Macrophage colony stimulating factor (MCSF) [known as (colony stimulating factor-1, CSF-1)] is responsible for the survival, proliferation and differentiation of mononuclear phagocytes through binding to M-CSFR [19]. Although IL-34 shares no apparent sequence homology with MCSF, the biological activity of IL-34 is mediated via interaction with M-CSFR, which is mainly expressed on the surface of macrophages [13].

We have previously demonstrated that IL-34 is substantially suppressed in gastric cancer and that IL-34 is an independent biomarker for predicting the development of gastric cancer [14]. Furthermore, it has been suggested that IL-34 may be involved in the development of HBV-HCC, using bioinformatic analysis in nude mice inoculated with human HCC, via manipulation of miR-28-5p [20]. Depletion of miR-28-5p has been demonstrated to enhance the progression of HCC, in combination with IL-34 and TAM in the HCC inoculated nude mice [20]. However, the role of IL-34 remains to be explored during the development of HBV-HCC in human in vivo. More recently, the correlation between IL-34 and MCSF in liver injury has been reported in chronic hepatitis C (CHC) patients with high fibrosis scores [12]. Additionally, circulating IL-34 has been associated with inflammatory activity and liver fibrosis in chronic hepatitis B (CHB) patients [21].

Serum tumor biomarkers are routinely used for surveillance and diagnosis of HCC, due to the non-invasive nature with relative objective and reproducible quantification [22]. Serum alpha-fetoprotein (AFP) is the most commonly referenced biomarker for the auxiliary diagnosis of HCC [22, 23]. However, AFP has ~ 10% false negative, due to its sensitivity in some early-stage or even a few late-stage HCC cases [23]. In an attempt to enhance both sensitivity and specificity in the accuracy of early diagnosis, imaging techniques are being developed including computed tomography (CT) or magnetic resonance imaging (MRI) [22]. The main limitation of CT and/or MRI is high operating cost, and shortage of sufficient competent technicians and/or specialists, which is increasingly challenging in rural regions without adequate financial support [22]. The development of an alternative method(s) with improved sensitivity and specificity for diagnosis of HBV-HCC patients, particularly of non-invasive nature, is therefore required to improve patient outcome. We have illustrated that the combination of inflammatory score/liver function and AFP improves the diagnostic accuracy of HBV-related HCC [24]. However, it still unclear if the combination of AFP and IL-34 could improve the sensitivity and specificity of HCC.

We hypothesize that IL-34 plays a critical role during the development of HBV-HCC, perhaps in conjunction with MCSF and TAM. This proposition is based on the close correlation between the severity of hepatic fibrosis and the incidence of HCC in HBV and/or hepatitis C viral (HCV) infected patients [25], as well as the information from HCC inoculated nude mice [20]. Thus, it was explored that the correlation among IL-34, MCSF and TAMs during the development of HBV-HCC at the different stages, particularly, the kinetics of IL-34 during the progression/development of HBV related liver diseases. In addition, the accuracy of diagnostic value of the combination of AFP and IL-34 with its related TAMs in HCC was investigated. Our current finding may be useful in the development of novel diagnostic and potential therapeutic target for the management of such devastating disease.

## Materials and methods

### Study population

Patients were identified between April 2015 and July 2017 in Department of Infectious Diseases, Shanghai Ruijin Hospital. Serum and liver tissues were obtained from the patients with informed consents. Healthy controls (HCs) were selected that age and sex matched healthy individuals for routine health check in our hospital without liver disease/HBsAg negative/negative image in CT or MRI. The selection of treatment for HBV-HCC patients was based on the guideline for treatment of primary liver cancer in China and was conducted with a multidisciplinary diagnosis and treatment team of Ruijin Hospital, as described previously [26]. HBV-HCC patients selected for the current study received either trans-hepatic arterial chemoembolization (TACE) or curative resection treatment. TACE, an interventional treatment, is one of the most common nonsurgical treatments for liver cancer [26]. Curative resection is a surgical procedure that hepatocellular cancerous tissue and a certain amount of normal tissue to be removed to obtain adequate margins. The purpose is to minimize the risk of any cancer cells being left behind.

The first inclusion criteria of CHB patients were: adult with consecutive HBsAg^+^ for at least six months, nucleos(t)ide (NA)-naïve without cirrhosis or carcinoma. The second inclusion criteria of HBV-HCC were: (1) Adult with consecutive HBsAg^+^ for at least six months; (2) Diagnosed as primary HCC confirmed with pathology; (3) AFP > 400 μg/L, no other active liver disease, pregnancy, embryonic source sex reproductive system tumor and metastatic liver cancer and could touch a swelling or hard of the liver with tumor, or imaging examination, such as computerized tomography (CT), magnetic resonance imaging (MRI) scans and ultrasound examinations, revealed liver occupying lesions characteristic; (4) AFP ≤ 400 μg/L, more than two imaging examinations revealed liver occupying lesions which has characteristic of HCC. The final inclusion criteria of HBV related cirrhosis (HBV-cirrhosis) were: (1) adult with consecutive HBsAg^+^ for at least six months; (2) diagnosed cirrhosis by biopsy of the liver; (3) or imaging examination, such as CT, MRI, FibroScan or ultrasound examinations, detected enlarged livers, abnormally nodular livers, enlarged spleens and fluid in the abdomen, suggesting cirrhosis; (4) the hospitalized patients under any event can also be diagnosed as decompensation liver cirrhosis: abdominal cavity effusion, esophageal gastric varices burst out of the blood, hepatic encephalopathy, infection.

The exclusion criteria were: (1) Co-infected with human immunodeficiency virus (HIV), hepatitis A virus (HAV), hepatitis C virus (HCV), hepatitis D virus (HDV) or hepatitis E virus (HEV); (2) Undergone liver transplantation before the study; (3) autoimmune liver disease, non-alcoholic fatty liver disease, alcoholic fatty liver disease, Wilson's disease or hemochromatosis; (4) pregnant women or breast-feeding; (5) liver metastatic tumors; (6) CHB related acute-on-chronic liver failure (ACLF). The exclusion criteria of healthy people were: (1) Undergone liver disease before the study; (2) Had abnormal liver function recently; (3) alcoholism (amount of alcohol: female ≥ 20 g/d, male ≥ 30 g/d).

This study complies with the declaration of Helsinki, and the study protocol (2018-141 Ruijin Hospital) was approved by the *Human Ethics Committee, Ruijin Hospital*. Written informed consent was obtained from all patients according to standards of the local ethics committees.

### Routine biochemistry and cytokine quantification

Routine biochemistry [alanine aminotransferase (ALT), aspartate aminotransferase (AST), alkaline phosphatase (AKP), gamma-glutamyl transpeptidase (r-GT), total bilirubin (TBil), albumin (Alb) and prothrombin time (PT)] and virologic tests [HBV-DNA level, hepatitis B surface antigen (HBsAg), hepatitis B surface antibody (anti-HBs), hepatitis B envelop antigen (HBeAg), hepatitis B envelop antibody (anti-HBe)] was performed. HBV-DNA < 5 * 10^2^ IU/mL was defined as low HBV-DNA, whereas, ≥ 5 * 10^2^ IU/mL was defined as high HBV-DNA [27]. HBeAg^+^ is defined as positive hepatitis B envelop antigen, and HBeAg^−^ is defined as negative hepatitis B envelop antigen. The Scheuer's scoring system was applied for pathology diagnosis of inflammation and fibrosis grading of liver tissue. Liver cirrhosis is defined as ≥ S4 of Scheuer's scoring system. Serum ALT, AST, AKP, r-GT, TBil and Alb (reflecting liver function) were quantified using Beckman coulter AU5800 automatic biochemical analyzer. HBsAg, anti-HBs, HBeAg and anti-HBe were determined using commercial ELISA kits (Abbott Diagnostics, IL). Serum HBV DNA levels were measured using qPCR, Roche Amplicor (Roche Diagnostic Systems, Branchburg, NJ, USA). Serum IL-34 and MCSF were quantified using ELISA (R&D Systems, Lille, France). Results were expressed as a concentration of cytokine production.

### Immunohistochemistry (IHC)

The liver tissue blocks were obtained from surgery for HBV-HCC (*n* = 30), liver biopsy for CHB liver (*n* = 5) or HBV-cirrhosis liver (*n* = 5) and the off cuts of liver transport donors for HCs (*n* = 5). HCs did not present liver disease/HBsAg negative/negative image in CT or MRI. Hepatic IL-34, MCSF and CD68 were determined using immunohistochemistry (IHC), using 3,3′-diaminobenzidine (DAB) color development. The primary antibodies were polyclonal rabbit anti-human IL-34 (bs-18170R, Beijing Biosynthesis Biotechnology, China), polyclonal rabbit anti-human MCSF (Abcam, Cambridge, UK) and monoclonal mouse anti-human CD68 (Dako, Copenhagen, Denmark). The sheep anti-rabbit conjugated HRP secondary antibody (Beijing Sequoia Jinqiao Biological Technology) was used. The specific target(s) was visualized with DAB detection kit and counterstained with hematoxylin. The IHC was repeated twice. Negative control was applied in each labeling for every primary rabbit negative control. Intra-hepatic IL-34 or MCSF is localized in the cytoplasm of hepatocytes, which has been well documented in our previous publications [28]. IHC was quantified using a computer-assisted genuine color image analysis system (ImagePro-plus 9.0) for hepatic IL-34, MCSF or CD68, as described previously [28, 29].

### Statistical analysis

Continuous variables were expressed as means ± standard deviation or median (inter-quartile range) where appropriate. Differences between two groups were determined by unpaired t-test or the Mann–Whitney U test. Among three groups were used by analysis of variance (ANOVA) or the Kruskal–Wallis H nonparametric test. Chi-square or Fisher’s exact test was employed to compare nominal variables. Correlations between variables were analyzed by Spearman’s correlation. ROC curve and binary logistic regression analysis were used for detecting the diagnostic accuracy of serum IL-34 or MCSF for HBV-HCC. All statistical tests are two-side, and *p* < 0.05 was considered to be statistically significant. SPSS version 22.0 was used for all statistical analysis (SPSS Inc., Chicago, IL, USA).

## Results

Baseline characteristics of patients were summarized in Table 1, HBV-HCC (*n* = 88), CHB (*n* = 64), HBV-cirrhosis (*n* = 64) and HCs (*n* = 20), according to the inclusion criteria.

**Table 1:** Demographic, clinical characteristic, biochemical characteristic

	HC (*n* = 20)	CHB (*n* = 64)	LC (*n* = 64)	HCC (*n* = 88)	*p* value
Age (mean ± SD)	66 ± 7	38 ± 12	48 ± 12	54 ± 8	< 0.0001
Sex					
Male	3/20 (15%)	44/64 (68.75%)	51/64 (79.69%)	82/88 (93.18%)	< 0.05
Female	17/20 (85%)	20/64 (31.25%)	13/64 (20.31%)	6/88 (6.82%)	< 0.05
ALT (IU/L)	19.7 ± 5.68	138.23 ± 191.06	162.54 ± 352.27	72.10 ± 80.05	< 0.05
AST (IU/L)	22.45 ± 4.5	78.03 ± 121.53	129.17 ± 277.76	102.20 ± 113.52	ns
AKP (IU/L)	66.55 ± 13.38	74.53 ± 24.31	97.33 ± 52.99	146.12 ± 98.07	< 0.001
r-GT (IU/L)	23.8 ± 13.35	54.33 ± 58.55	83.32 ± 103.52	129.68 ± 117.82	< 0.001
TBil (μmol/L)	12.61 ± 4.00	21.50 ± 15.67	54.58 ± 86.55	65.69 ± 87.12	< 0.001
Alb (g/L)	–	42.44 ± 9.06	35.97 ± 8.86	32.54 ± 7.00	< 0.001
PT (s)	–	12.48 ± 1.61	14.06 ± 2.44	13.95 ± 2.13	< 0.001
AFP (μg/L)	–	36.45 ± 137.73	49.14 ± 125.28	2319.21 ± 5558.19	< 0.0001
HBeAg					< 0.05
HBeAg^−^	–	28/64 (43.75%)	34/63 (53.97%)	39/57 (68.42%)	
HBeAg^+^	–	36/64 (56.25%)	29/63 (46.03%)	18/57 (31.58%)	
HBV-DNA (IU/ml)					< 0.001
< 5*10^2^	–	11/63 (17.46%)	20/60 (33.33%)	35/52 (67.31%)	
≥ 5*10^2^, ≤ 10^4^	–	17/63 (26.98%)	14/60 (23.33%)	8/52 (15.38%)	
> 10^4^	–	35/63 (55.56%)	26/60 (43.33%)	9/52 (17.31%)	
IL-34 (pg/ml)	15.71 ± 4.74	21.22 ± 7.17	26.58 ± 15.83	35.74 ± 27.85	< 0.05
MCSF (pg/ml)	161.14 ± 146.32	134.66 ± 138.68	119.66 ± 78.98	238.31 ± 516.30	ns

ALT, alanine aminotransferase; AST, aspartate aminotransferase, AKP, alklinephosphatase; r-GT, gamma-glutamyl transpeptidase; TBil, total bilirubin; Alb, albumin; PT, prothrombin time; BCLC, Barcelona Clinic Liver Cancer; LC, liver cirrhosis

### Serum IL-34 and MCSF were elevated in HBV-HCC

Significant differences of biochemical indices (ALT, AKP, GGT, TBil, PT and AFP) were identified among CHB, HBV-cirrhosis and HBV-HCC patients, except AST (Table 1). The levels of AFP were significantly higher in HBV-HCC than CHB, HBV-cirrhosis (*p* < 0.01). AFP was 36.45, 49.14 or 2319.21 μg/l from CHB, HBV-cirrhosis or HBV-HCC patients, respectively. Serum IL-34 and MCSF were elevated in HBV-HCC.

**Fig. 1:**
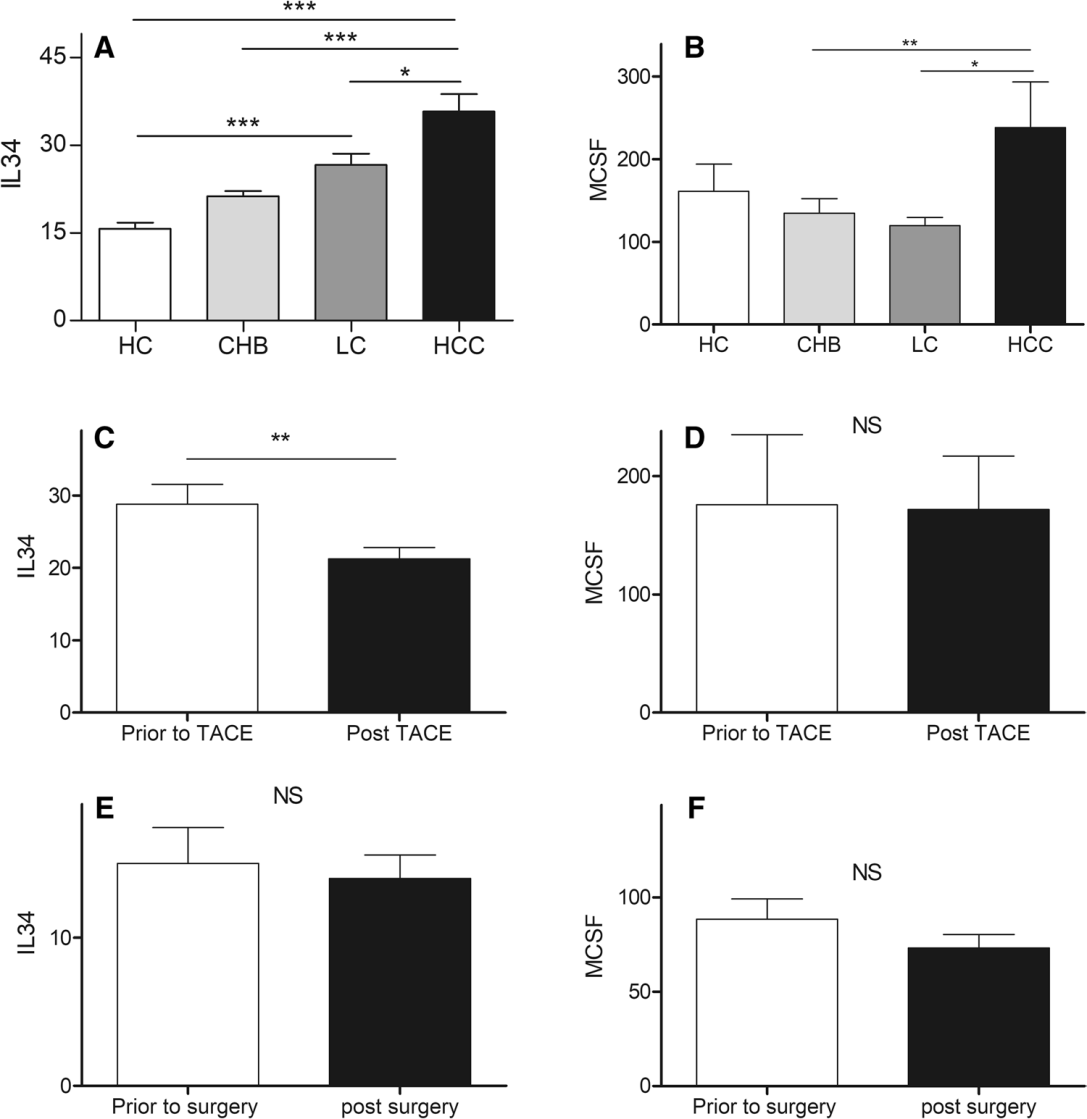
**A** Serum IL-34 of HCs, CHB, HBV-cirrhosis and HBV-HCC; **B** Serum MCSF of HCs, CHB, HBV-cirrhosis and HBV-HCC. (LC: liver cirrhosis); **C** The serum IL-34 in HBV-HCC patients prior to and post-trans-hepatic arterial chemotherapy and embolization (TACE); **D** Serum MCSF in HBV-HCC patients prior to and post-TACE; **E** Serum IL-34 in HBV-HCC patients prior to and post-surgery; **F** Serum MCSF in HBV-HCC patients prior to and post-surgery

Serum IL-34 was 1.7, 1.3 or 2.3-fold higher from HBV-HCC groups than that from CHB (35.74 vs. 21.22, *p* < 0.01), HBV-cirrhosis (35.74 vs. 26.58, *p* < 0.05) or HCs (35.74 vs. 15.71, *p* < 0.01) (Table 1; Fig. 1A). Serum MCSF was 1.8 or 2.0-fold higher from HBV-HCC groups than that from CHB (238.3 vs. 134.7, *p* < 0.01) or HBV-cirrhosis (238.3 vs. 119.7, *p* < 0.05). However, there was no significant difference of serum IL-34 between HBV-cirrhosis patients and HCs (26.58 vs. 21.22, *p* > 0.05), nor between CHB patients and HCs (21.22 vs. 15.71, *p* > 0.05). In addition, there was no significant difference of serum MCSF between HCs and CHB, HBV-cirrhosis or HBV-HCC groups (Table 1; Fig. 1B).

### Serum IL-34 or MCSF during anti-tumor treatment in HBV-HCC

We further determined the serum IL-34 or MCSF in HBV-HCC patients received TACE or surgery, using ELISA. Serum IL-34 was decreased significantly by > 20% post-TACE in HBV-HCC patients, compared to that prior to the treatment (28.82 vs. 21.23, *p* < 0.01) (Fig. 1C); No significant difference of MCSF was observed in the HBV-HCC patients between prior to and post-TACE treatment (175.6 vs. 171.7, *p* > 0.05) (Fig. 1D). In addition, in HBV-HCC patients undergoing surgery, there was no significant change of circulating IL-34 (15.01 vs. 14.01, *p* > 0.05) (Fig. 1E) and MCSF (88.32 vs. 73.23; *p* > 0.05) (Fig. 1F) between prior to and post-surgery.

### The factors that are associated with HBV-HCC incidence

Correlation between IL-34 or MCSF and incidence of HBV-HCC, as well as other HBV related influence factor, including ALT, AST, HBV-DNA, HBsAg, HBeAg, AFP, were analyzed using Spearman rank correlation (Table 2). Serum IL-34 was positively correlated with the incidence of HBV-HCC (*r*_s_ = 0.257, *p* < 0.01), as well as, MCSF (*r*_s_ = 0.223, *p* < 0.01) and AFP (*r*_s_ = 0.525, *p* < 0.01). HBsAg, HBeAg or HBV-DNA were inversely correlated with HBV-HCC (*r*_s_ = − 0.441, *p* < 0.01; *r*_s_ = − 0.557, *p* < 0.01; or *r*_s_ = − 0.428, *p* < 0.01, respectively). There was no significant correlation between serum IL-34 and other HBV related influence factor, including ALT, AST, HBV-DNA, HBsAg, AFP (Supplement Fig. 1).

**Table 2:** Factors associated with the incidence of HBV-HCC

Marker	HBV-HCC	
	r_s_	*p* value
IL-34 (pg/ml)	0.257	< 0.01
MCSF (pg/ml)	0.223	< 0.01
ALT (IU/L)	− 0.109	ns
AST (IU/L)	0.190	< 0.01
AKP (IU/L)	0.428	< 0.01
GGT (IU/L)	0.346	< 0.01
TB (μmol/L)	0.324	< 0.01
Alb (g/L)	− 0.425	< 0.01
PT (s)	0.182	< 0.05
AFP (μg/L)	0.525	< 0.01
HBsAg	− 0.441	< 0.01
HBeAg	− 0.557	< 0.01
HBV-DNA (IU/ml)	− 0.428	< 0.01

**Fig. 2:**
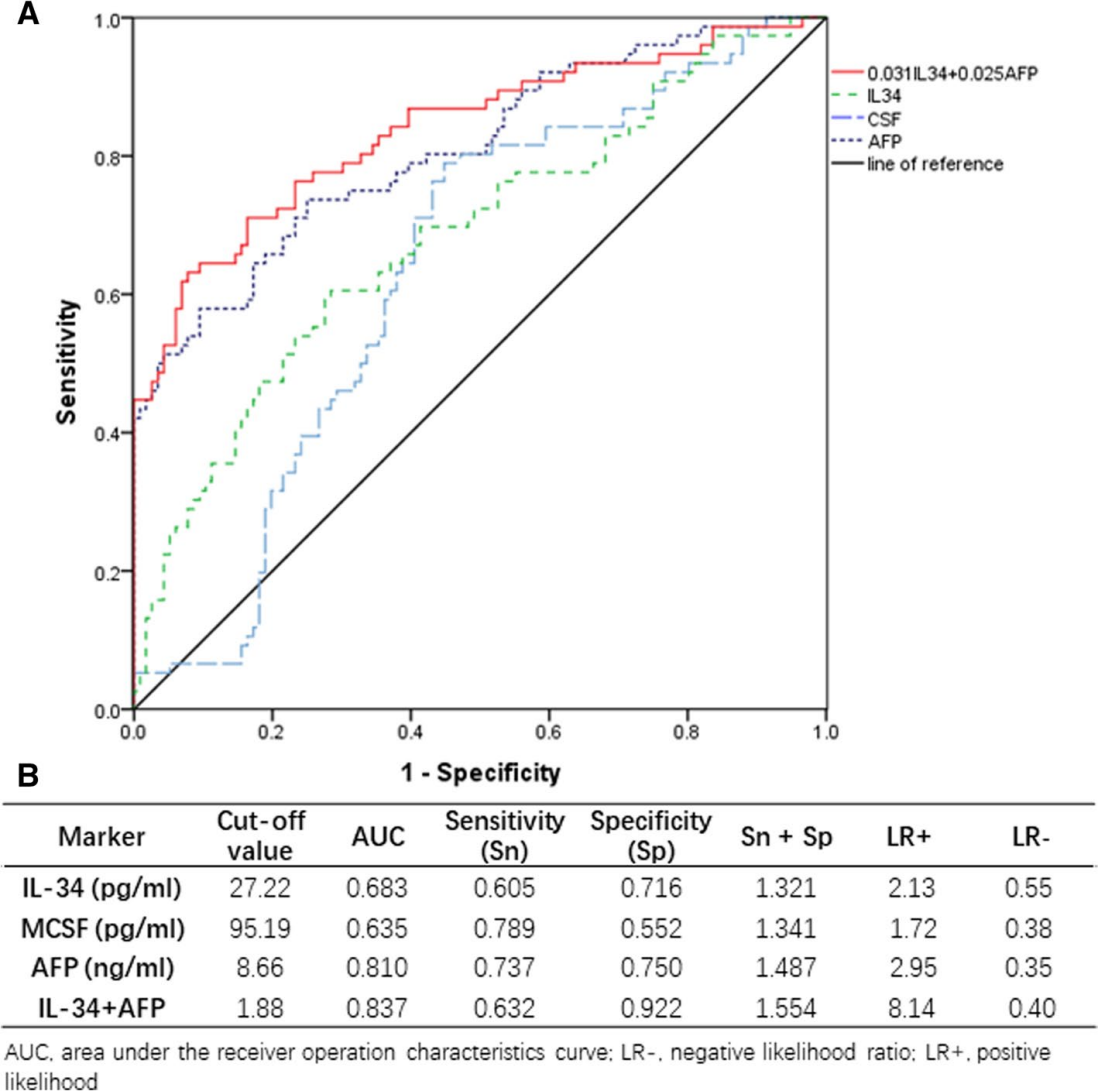
ROC curves of AFP, IL-34, MCSF and AFP combined IL-34 diagnosis for HBV-HCC

Diagnostic accuracy of serum IL-34 or MCSF for detecting HBV-HCC was further determined using ROC curve. AUC, sensitivity, specificity, LR+ and LR− for serum IL-34, MCSF or AFP for diagnosing HBV-HCC are shown in Fig. 2B. AUC values for serum IL-34, MCSF or AFP were 0.683 (0.605–0.761), 0.635 (0.556–0.714) or 0.810 (0.746–0.874), respectively (Fig. 2). Considering the limited sensitivity or specificity of the three markers, AFP was combined with serum IL-34 and MCSF for detecting HBV-HCC. Moreover, AFP plus serum IL-34 showed the highest AUC (0.837) with sensitivity (0.632) and highest specificity (0.922). MCSF could not be combined with other two factors. Based on these parameters and binary logistic regression analysis, the final equation was established: *Y* = 0.031 × IL-34 + 0.025 × AFP, which could be used for prediction of HBV-HCC among CHB patients. To determine if IL-34 combined with AFP could improve the specificity in determining HCC, i.e., tumor size, we compared IL-34 versus tumor size, AFP vs tumor size or IL 34 + AFP versus tumor size (Supplementary Fig. 2). There was no significant correlation between IL-34 and tumor size. As a standard control, a significant correlation between AFP and tumor size was observed. Additionally, a significant correlation was observed between AFP and tumor size or IL 34 + APF and tumor size, based on univariable analysis. Furthermore, there was substantially more area of ROC from APF + IL-34 in predicting the incidence of HCC, compared to that from AFP alone, despite similar observed patterns between IL-34 + AFP vs tumor size, and AFP vs tumor size.

### Intra-hepatic IL-34 from CHB, HBV-cirrhosis, HBV-HCC patients

**Fig. 3:**
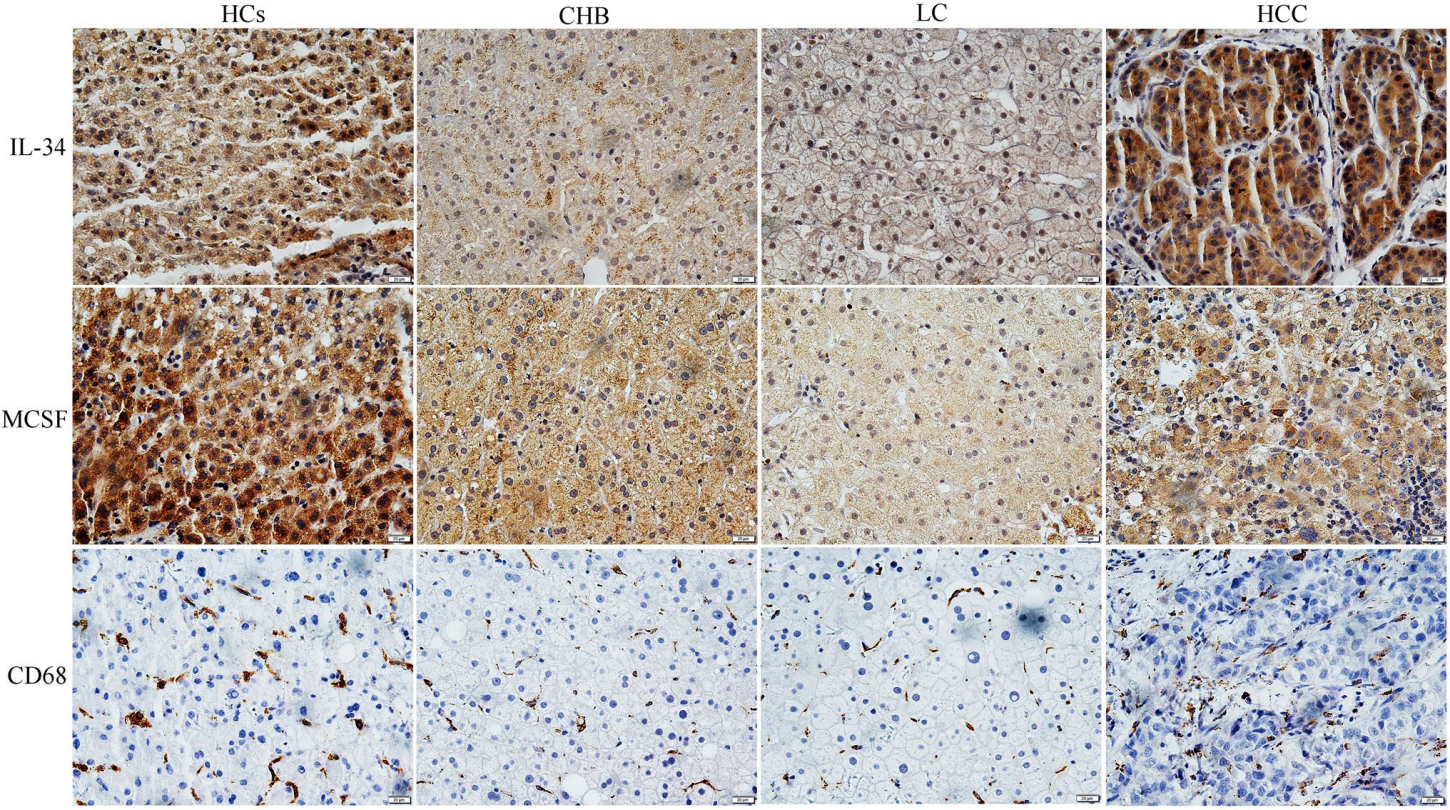
The immunohistochemistry of intra-hepatic IL-34, MCSF, CD68^+^TAMs in HCs, CHB, HBV-cirrhosis and HBV-HCC (LC: liver cirrhosis)

**Fig. 4:**
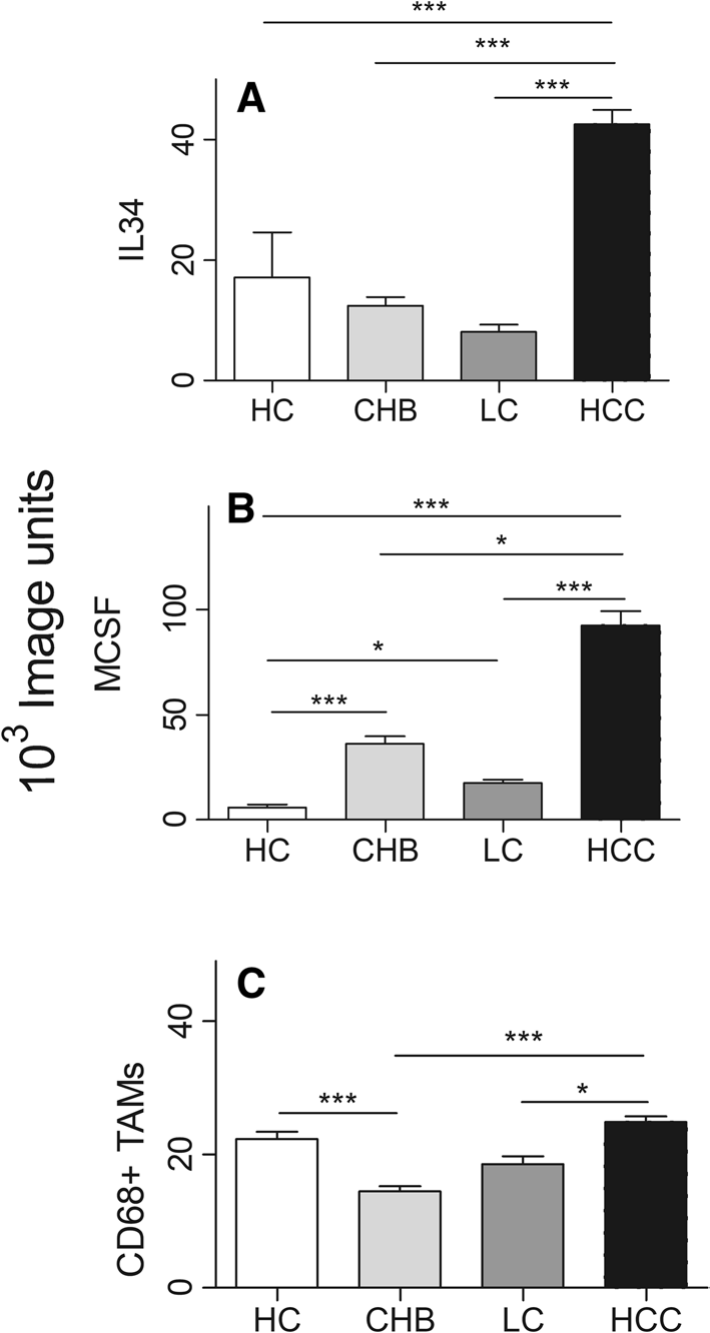
The corresponding quantification of immunohistochemical detection (LC: liver cirrhosis)

According to the inclusion criteria, specimens of liver tissue were obtained from HBV-HCC (*n *= 30), CHB patients (*n* = 5), HBV-cirrhosis (*n* = 5), HCs (*n* = 5) (Supplement Table 1). There was no significantly difference of biochemical indices (ALT, AST, AKP, GGT, TBil, PT and AFP) among CHB, HBV-cirrhosis and HBV-HCC patients, except Alb (Supplement Table 1). Intra-hepatic IL-34 and MCSF from HBV-HCC patients were significantly higher than that of CHB, HBV-cirrhosis and HCs (Figs. 3, 4) (*p* < 0.05). Intra-hepatic CD68^+^ TAMs were increased 1.7 or 1.3-fold in HBV-HCC, compared to that from CHB or HBV-cirrhosis, respectively (Fig. 4). No significant difference of intra-hepatic CD68^+^ TAMs was observed between HBV-HCC patients and HCs.

### Correlation between intra-hepatic IL-34, MCSF and CD68+ TAMs in HBV-HCC and clinical parameters

**Fig. 5:**
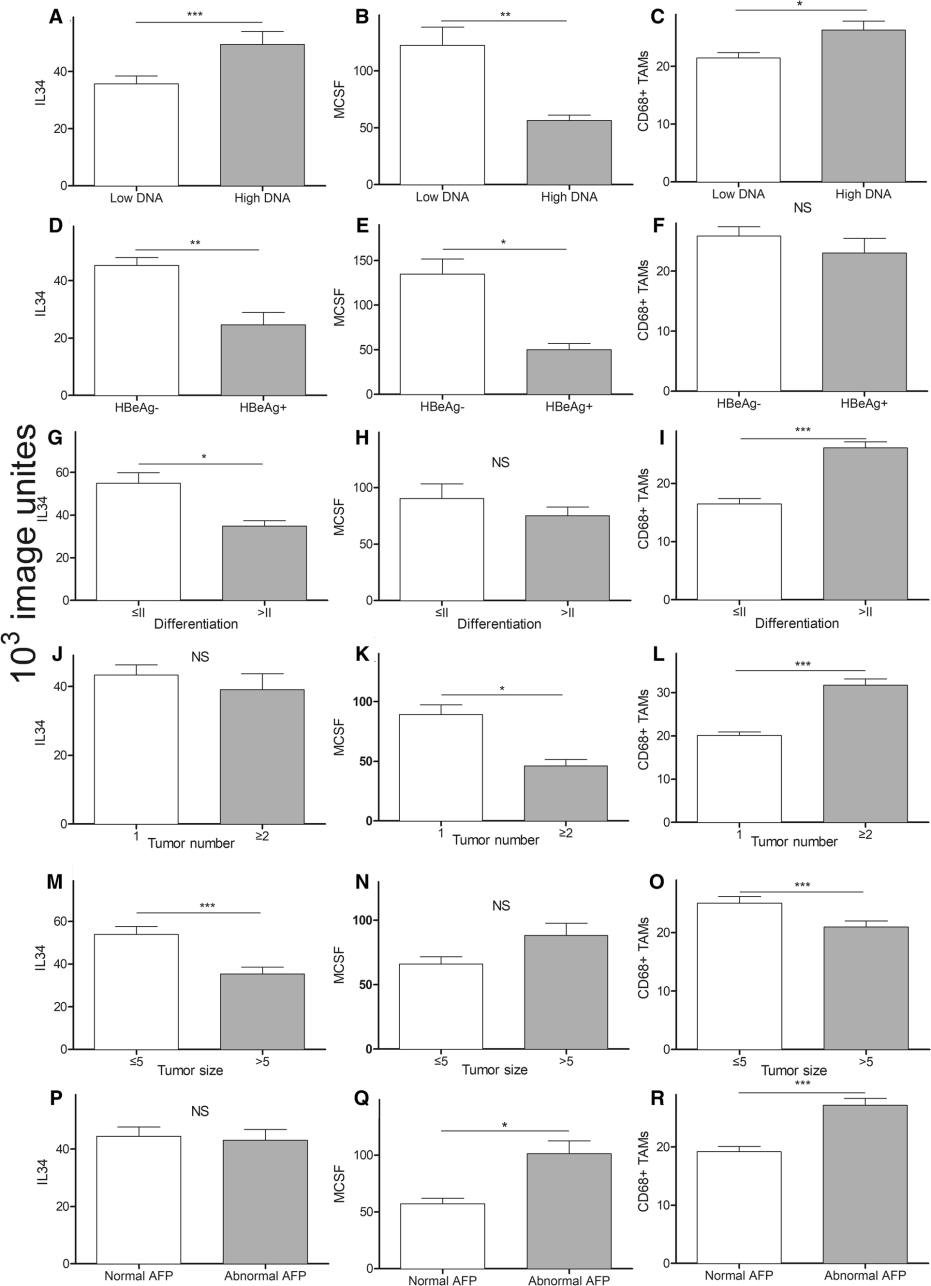
Correlation of intra-hepatic IL-34, MCSF or CD68^+^ TAMs expression with HBV-DNA, HBeAg and tumor differentiation subtypes. **A** Correlation of intra-hepatic IL-34 with HBV-DNA; **B** Correlation of intra-hepatic MCSF with HBV-DNA; **C** Correlation of intra-hepatic CD68^+^ TAMs expression with HBV-DNA; **D** Correlation of intra-hepatic IL-34 with HBeAg; **E** Correlation of intra-hepatic MCSF with HBeAg; **F** Correlation of intra-hepatic CD68^+^ TAMs expression with HBeAg; **G** Correlation of intra-hepatic IL-34 with tumor differentiation; **H** Correlation of intra-hepatic MCSF with tumor differentiation; **I** Correlation of intra-hepatic CD68^+^ TAMs expression with tumor differentiation; **J** Correlation of intra-hepatic IL-34 with tumor number; **K** Correlation of intra-hepatic MCSF with tumor number; **L** Correlation of intra-hepatic CD68^+^ TAMs expression with tumor number; **M** Correlation of intra-hepatic IL-34 with tumor size; **N** Correlation of intra-hepatic MCSF with tumor size; **O** Correlation of intra-hepatic CD68^+^ TAMs expression with tumor size; **P** Correlation of intra-hepatic IL-34 with AFP; **Q** Correlation of intra-hepatic MCSF with AFP; **R** Correlation of intra-hepatic CD68^+^ TAMs expression with AFP

Associations between clinical pathological parameters of HBV-HCC and intra-hepatic IL-34, MCSF or CD68^+^ TAMs were summarized in Table 3, Supplement Table 2. IL-34 was associated with HBV-DNA, HBeAg, tumor differentiation and tumor size of HBV-HCC patients (Fig. 5). IL-34 was 28% lower in the group of patients with low HBV-DNA level compared to patients with high level (*p* < 0.05). Nearly 50% reduced intra-hepatic IL-34 was also observed in HBeAg^+^ compared to HBeAg^−^ HBV-HCC patients (*p* < 0.05). In addition, significant inverse correlation was observed between IL-34 and differentiation or tumor size of HCC (Fig. 5). IL-34 was increased by 1.3-fold in low differentiated HCC compared to that of high differentiation group (*p* < 0.05); as well as, 1.3-fold in intra-hepatic IL-34 production from small tumor size (≤ 5 cm) than that from big size tumor group (*p* < 0.05). However, there was no correlation between IL-34 and other parameters, including tumor number and AFP of HCC. Intra-hepatic CD68^+^ TAMs were associated with high HBV-DNA, high tumor differentiation, small tumor size, abnormal AFP and increased tumor number. On the other hand, MCSF was inversely associated with HBV-DNA, HBeAg^−^, abnormal AFP and tumor number in HBV-HCC patients. Interestingly, MCSF does not associate with tumor differentiation or tumor size.

**Table 3:** Intra hepatic IL 34, MCSF or CD68^+^ TAMs in different clinical features of patients with HBV-HCC (*n* = 30)

Characteristics	*N*	IL-34		MCSF		CD68^+^ TAMs	
		Median	*p* value	Median	*p* value	Median	*p* value
HBV-DNA (IU/mL)							
< 5*10^2^	15	35.7	< 0.001	122.3	< 0.01	21.5	< 0.05
≥ 5*10^2^	11	49.4		56.3		26.3	
HBeAg							
HBeAg^−^	10	45.3	< 0.01	134.7	< 0.05	25.8	Ns
HBeAg^+^	3	24.6		50.0		23.0	
AFP							
Normal	14	44.4	Ns	57.2	< 0.05	19.2	< 0.0001
Abnormal	16	43.0		101.2		27.2	
Differentiation							
≤ II	10	54.9	< 0.05	90.3	ns	16.5	< 0.0001
> II	19	34.8		75.1		26.1	
Tumor number							
1	22	43.4	ns	89.1	< 0.05	20.1	< 0.0001
≥ 2	7	39.1		46.0		31.7	
Tumor size							
≤ 5	12	53.9	< 0.0001	66.0	ns	25.0	< 0.001
> 5	17	34.8		88.1		21.0	

All datum was *10^3^ image unites. The reference range of AFP is 0–9 μg/L. The histopathological classification is well described in the published Literature

## Discussion

In the present study, we evaluate circulating and intra-hepatic IL-34 in HBV related liver diseases. Circulating IL-34 of HBV-HCC patients was significantly higher than that of CHB, HBV-cirrhosis and HCs. The highest AUC was detected from AFP plus serum IL-34, suggesting the combination boosts sensitivity and specificity over AFP alone. Furthermore, circulating IL-34 was suppressed with anti-tumor TACE treatment in HBV-HCC, further confirming the potential role of IL-34 during the development of HBV-HCC. Consistent with circulating IL-34, upregulated intra-hepatic IL-34 from HBV-HCC was also detected, compared to that of CHB, HBV-cirrhosis and HCs. Intra-hepatic IL-34 was associated with high HBV-DNA, HBeAg^−^, poor tumor differentiation and small tumor size in HBV-HCC patients. Intra-hepatic CD68^+^ TAMs were upregulated in HBV-HCC compared to that from CHB and HBV-cirrhosis. Intra-hepatic CD68^+^ TAMs were associated with high HBV-DNA, high tumor differentiation, small tumor size, abnormal AFP and increased tumor number. Our data suggest that IL-34 contributes to the development of HBV-HCC, i.e., promoting the disease progression from CHB, HBV cirrhosis and then HBV-HCC. This observation is supported by findings from the Zhou group, showing that IL-34 is a key regulator for the growth of HCC in nude mice, via miR-28-5p mediated activation of TAM [20]. Our finding is, therefore, an extension and validation of the important role of IL-34 during the development of HBV-HCC in human tissues.

Our study identified circulating IL-34 from HBV-HCC patients was significantly higher than that of CHB, HBV-cirrhosis and HCs, suggesting that IL-34 may contribute to tumorigenesis of HCC, enhancing progression from CHB patients to cirrhosis, and finally HCC. Furthermore, intra-hepatic IL-34 expression was consistent with circulating IL-34, which is in line with previous studies, showing that high IL-34 in autoimmune diseases [15, 16]. More specifically, IL-34 is overexpressed in the inflamed synovium of rheumatoid arthritis patients, where it appears to act synergistic with TNF and IL-1β, inducing osteoclastogenesis and contributing to tissue inflammation and bone erosion [30]. In addition, upregulated circulating and intra-hepatic IL-34 in HBV-HCC from our current study is supported by the others, showing that the circulating IL-34 markedly increased in HBV-HCC patients, compared to those in CHB and HBV-negative HCC patients [31], and the HBx gene of HBV upregulates IL-34 expression in hepatoma [32].

However, our previous study demonstrates that IL-34 is inversely correlated with differentiation, metastasis and invasion of gastric cancer [14], which is rather controversial with our current discovery in HBV-HCC disease. Our explanation for such discrepancy between HBV-HCC and gastric cancer is more likely related to the carcinogenic differences between gastric cancer and HBV-HCC, as well as completely different micro-environments, despite of gastric cancer and HBV-HCC all belonging to the gastrointestinal system. In our current study, we observed that IL-34 decreased following TACE but not with surgery. This decrease may be attributed to TACE often being applied for liver cancer patients with multiple-space occupying lesions, usually with large tumor, whereas surgically removed liver cancers are often small. TACE (localized chemotherapy), therefore, effectively induces malignant cellular damage inside the liver cancer, subsequently down-regulating HCC cellular function, including IL-34 production [32]. In contrast, surgical resection is removing smaller space occupying lesion than to these with TACE patients, thus relative less effect on the liver, particularly HCC cellular function.

Tumor development is closely related to the micro-environment, including tumor cells, monocytes/macrophages, cytokines and neovascularization. TAMs are mixed phenotype, expressing M1 or M2 markers [5], and may be influenced by different microenvironments in different regions and/or in different individuals. In our current study, intra-hepatic CD68^+^ TAMs increased gradually with the order from CHB, HBV-cirrhosis to HBV-HCC patients. Thus, we speculate that the increased infiltrating CD68^+^ TAMs may be M2 dominant, contributing to stimulate tumor growth activity [5]. This speculation is in line with Zhou et al. showing that IL-34 induced TAMs can inhibit miR-28-5p in HCC cells in vitro via TGFβ1, suggesting an interaction among miR-28-5p, IL-34 and macrophage [20]. In clinical HCC study, lower miR-28-5p is correlated with high IL-34 and TAMs in HCC patients with a poor overall survival and recurrence [20].

IL-34 is upregulated in HCV infection and inhibited the production of IFN-γ [12]. IL-34 may also be associated with inflammatory activity and liver fibrosis in CHB [21]. Moreover, baseline circulating IL-34 has been shown to serve as a prognostic factor for progression in such patients [12, 21]. The high serum IL-34 level is associated with poor prognosis in non-viral HCC patients, compared to patients with low serum IL-34 level [31]. In our current study, only circulating IL-34 is significantly decreased in HBV-HCC patients post-anti-tumor treatment, compared to pre-treatment. These observations suggest that IL-34 is closely correlated with the weight of HBV-HCC or partial source of IL-34 is coming from HCC cells. Thus, serum IL-34 was significantly correlated with the incidence of HBV-HCC. The sensitivity and specificity of AFP combined with circulating IL-34 appears greatly improved, compared to that of AFP alone. These results suggest that AFP combined with serum IL-34 could improve the diagnostic accuracy of AFP for detecting HBV-HCC. Additionally, IL-34 could be used as a diagnostic biomarker for HBV-HCC, which will be further clarified in vitro and in vivo.

IL-34 induces differentiation of leukemia cells into mature macrophages [33], while additionally enhancing differentiation of other cancers [14, 34]. These reports are consistent with our current findings that intra-hepatic IL-34 expression correlates with the differentiation and tumor size of HBV-HCC. Our data demonstrate that the combination of IL-34 and AFP levels significantly correlates with HCC and is more sensitive and specific compared to AFP alone. Such data suggest that combined IL-34 and AFP is able to detect HCC at the relatively early stage with specificity, compared the conventional AFP or the relative new IL-34. The precise underlying mechanism of IL-34 and AFP involved in the development of HCC is currently being investigated.

Our data may provide an explanation for the possible role of IL-34 in the development of HBV-HCC, i.e., IL-34 regulates differentiation of HBV-HCC, which would have potential clinical relevance regarding IL-34 as a therapeutic target for malignancy. This speculation is in line with others, showing that IL-34 inhibits HBV replication in vivo and in vitro [35], and further supports that IL-34 is beneficially to the HBV-HCC patients for potential therapeutic target. Our current study demonstrates that intrahepatic IL-34 and TAMs are associated with high HBV-DNA, in addition to small tumor size of HBV-HCC patients. This is consistent with others, showing that HBx gene of HBV (part of HBV DNA) upregulates IL-34 expression in hepatoma [32]. Our explanation of the correlation among IL-34, TAMs and small tumor size of HCC, but not large tumor size, is such: IL-34 is highly produced by HCC cells from the small size HCC, supporting our finding that high IL-34 was detected in the liver from HCC, as well as from the circulation. However, there may be more disturbance at pathophysiological level in the large size HCC, which is particularly presented with some necrosis in the center of large HCC either due to nutritional competition or might be physical compression due to space limitation [36]. Thus, HCC cells from the large size tumor may not function properly, and consequently didn’t produce IL-34 as high as small tumor size patients correspondingly. We don’t have firm evidence to support this hypothesis. Nevertheless, our discussion provides logical explanation for our observation, which will be confirmed in our future experiment.

We acknowledge limitations in the current study. Kinetics of intra-hepatic or circulating IL-34 during the development and management of HCC were not performed. Correlation between IL-34 and prognosis was additionally not detected. These two interesting points will be determined in our future study. Otherwise, the correlation coefficients r listed between IL-34/MCSF and the incidence of HBV-HCC were (*r* = 0.257, 0.223), which was rather low, despite that *p* < 0.01. The low coefficient may be due to relatively small sample size of the current study, which may compromise certain level of conclusion. However, the current experiment does offer an objective observation for a proof of concept. We will explore the underlying mechanism with a large sample size and multiple center studies in future. We would additionally like to extend our research into multi-center study. However due to time constraints, including application for human ethical committee and establishment with collaborators, we are not able to complete this goal. We are current planning to verify and extend to explore the signaling pathway for our future study.

In conclusion, the current study improves our understanding of the role of IL-34 and AFP in HBV related liver disease. Increased IL-34 may contribute to the transformation of HBV-HCC, which is a potential predictor of HBV-HCC. The underlying mechanism of IL-34 in HBV-HCC is being currently investigated.

## Supplementary Information

Below is the link to the electronic supplementary material.

Supplementary file1 (DOCX 36 KB)

Supplement Figure 1. Correlation between serum IL-34 and other HBV related influence factors. (DOCX 644 KB)

Supplement Figure 2. Correlation between serum IL-34 and/or AFP and tumor size in HBV-HCC patient (DOCX 458 KB)

## Data Availability

All data generated or analyzed during this study were all included in this present article.

## Abbreviations

ACLF: Acute-on-chronic liver failure
AFP: Alpha-fetoprotein
AKP: Alkline phosphatase
ALT: Alanine aminotransferase
Alb: Albumin
ANOVA: Analysis of variance
anti-HBe: Hepatitis B envelop antibody
anti-HBs: Hepatitis B surface antibody
AST: Aspartate aminotransferase
CHB: Chronic hepatitis B
CHC: Chronic hepatitis C
CSF-1: Colony stimulating factor-1
CT: Computed tomography
HAV: Hepatitis A virus
HBV: Hepatitis B virus
HBV-HCC: Hepatitis B virus related hepatocellular carcinoma
HBV-cirrhosis: Hepatitis B virus related cirrhosis
HBeAg: Hepatitis B envelop antigen
HBsAg: Hepatitis B surface antigen
HCC: Hepatocellular carcinoma
HCs: Healthy controls
HCV: Hepatitis C virus
HDV: Hepatitis D virus
HEV: Hepatitis E virus
HIV: Human immunodeficiency virus
MCSF: Macrophage colony stimulating factor
M-CSFR: Macrophage colony stimulating factor receptor
MRI: Magnetic resonance imaging
PT: Prothrombin time
r-GT: Gamma-glutamyl transpeptidase
TACE: Trans-hepatic arterial chemoembolization
TAMs: Tumor associated macrophage
TBil: Total bilirubin
