# Supplementary material for: Combination of IL-34 and AFP improves the diagnostic value during the development of HBV related hepatocellular carcinoma
Source: Clin Exp Med. 2022 Mar 28;23(2):397–409. doi: 10.1007/s10238-022-00810-7 (PMC10224837; doi:10.1007/s10238-022-00810-7)
Supplement: Supplementary file 1 — Supplementary file1 (DOCX 36 KB) [file 10238_2022_810_MOESM1_ESM.docx]

**Supplement Table 1**. Baseline characteristics of the study population of immunohistochemistry.

|  | | CHB (n=5) | LC (n=5) | HCC (n=30) | *p* value |
| --- | --- | --- | --- | --- | --- |
| Age (mean ± SD) | | 35.40 ± 5.41 | 49.20 ± 6.46 | 59.43 ± 14.71 | <0.01 |
| Sex | Male | 4/5 (80%) | 3/5 (60%) | 26/30 (86.67%) | <0.05 |
|  | Female | 1/5 (20%) | 2/5 (40%) | 4/30 (13.33%) | <0.05 |
| ALT (IU/L) | | 69.00 ± 64.82 | 73.20 ± 60.32 | 44.67 ± 55.48 | ns |
| AST (IU/L) | | 43.50 ± 33.13 | 47.80 ± 33.21 | 208.33 ± 927.59 | ns |
| AKP (IU/L) | | 64.00 ± 26.67 | 68.60 ± 8.91 | 76.03 ± 35.93 | ns |
| r‑GT (IU/L) | | 31.50 ± 21.38 | 51.60 ± 37.10 | 55.40 ± 45.34 | ns |
| TBil (μmol/L) | | 15.05 ± 3.96 | 10.58 ± 5.40 | 16.68 ± 8.96 | ns |
| Alb (g/L) | | 43.50 ± 3.51 | 44.40 ± 1.14 | 36.77 ± 6.73 | <0.05 |
| PT (s) | | 12.05 ± 1.42 | 12.06 ± 0.83 | 11.87 ± 0.91 | ns |
| AFP (μg/L) | | 4.95 ± 3.42 | 15.38 ± 22.77 | 1577.71 ± 3349.61 | ns |
| HBV-DNA (IU/ml) | <5*10^2^ | 1/5 (20%) | 3/5 (60%) | 16/26 (61.5%) | ns |
|  | ≥5*10^2^ | 4/5 (80%) | 2/5 (40%) | 10/26 (38.5%) |  |

**Supplement Table 2.** Correlation between intra-hepatic IL-34/MCSF/CD68+ TAMs and clinical pathological parameters of HBV-HCC.

| Characteristics | IL-34 | | | | MCSF | | | | CD68^+^ TAMs | | | |
| --- | --- | --- | --- | --- | --- | --- | --- | --- | --- | --- | --- | --- |
|  | r_s_ | | *p* value | | r_s_ | | *p* value | | r_s_ | | *p* value | |
| IL-34 | |  | |  | | 0.317 | | ns | | 0.491 | | <0.01 |
| MCSF | | 0.317 | | ns | |  | |  | | 0.517 | | <0.01 |
| Differentiation | | 0.187 | | ns | | -0.092 | | ns | | -0.305 | | ns |
| Tumor number | | -0.087 | | ns | | -0.177 | | ns | | 0.418 | | <0.05 |
| Tumor size (cm) | | -0.248 | | ns | | 0.032 | | ns | | -0.06 | | ns |
