# Supplementary figures and images for: Combination of IL-34 and AFP improves the diagnostic value during the development of HBV related hepatocellular carcinoma
Source: Clin Exp Med. 2022 Mar 28;23(2):397–409. doi: 10.1007/s10238-022-00810-7 (PMC10224837; doi:10.1007/s10238-022-00810-7)

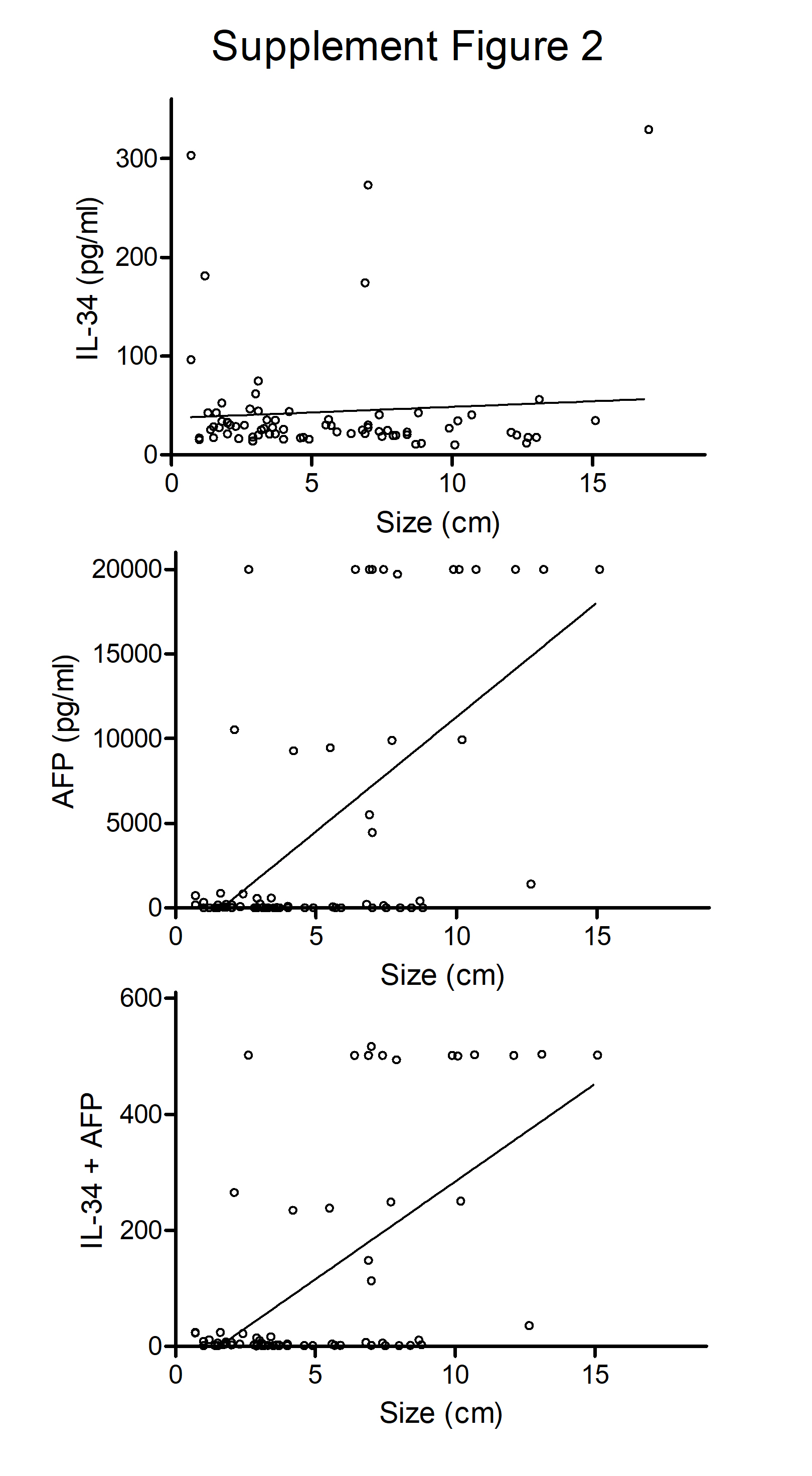

Supplement: Supplementary file 2 — Supplement Figure 1. Correlation between serum IL-34 and other HBV related influence factors. (DOCX 644 KB) [file 10238_2022_810_MOESM2_ESM.jpg]

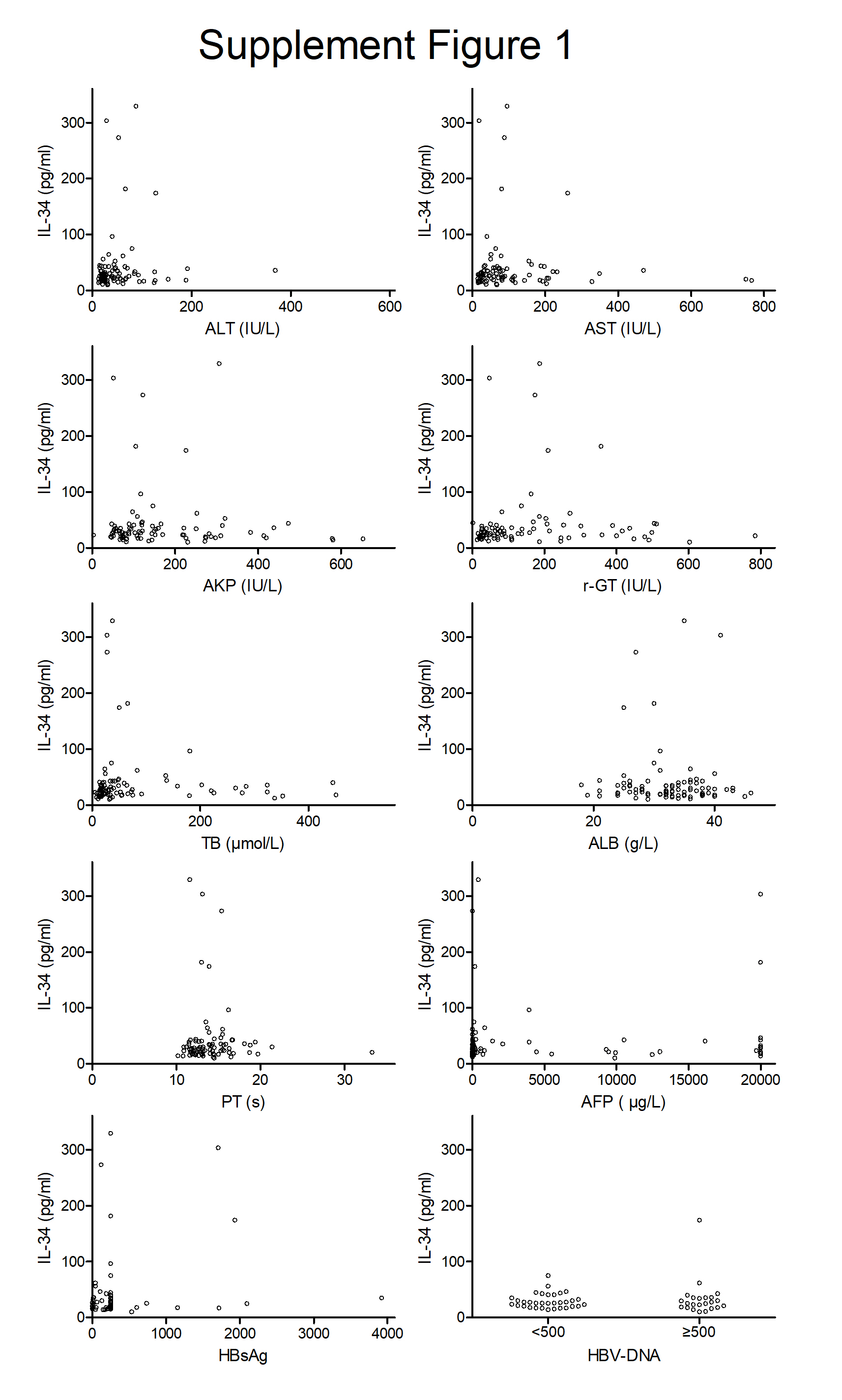

Supplement: Supplementary file 3 — Supplement Figure 2. Correlation between serum IL-34 and/or AFP and tumor size in HBV-HCC patient (DOCX 458 KB) [file 10238_2022_810_MOESM3_ESM.jpg]
